# Hemolytic Disease of the Fetus and Newborn Due to Anti-Gonzales Antibody

**DOI:** 10.7759/cureus.36860

**Published:** 2023-03-29

**Authors:** Kaila R Fives, Danielle A Chism, Bailey Beetz, Isaac Elkins, Madhura Butala

**Affiliations:** 1 School of Medicine, Lake Erie College of Osteopathic Medicine, Bradenton, USA; 2 Pediatrics, Ascension St. Vincent's, Jacksonville, USA

**Keywords:** rh alloimmunization, abo and rh blood groups, coombs positive hemolysis, anemia and hyperbilirubinemia, hemolytic disease of the fetus and newborn

## Abstract

Hemolytic disease of the fetus and newborn (HDFN) is an immune-mediated condition caused by the production of maternal antibodies to fetal red blood cells. This condition most commonly arises due to Rh factor incompatibility. The case presented here displays an example of HDFN in which the mother and fetus's blood type was O+. Upon further investigation, it was determined that the mother is a producer of anti-Gonzales antibodies (anti-Go(a)). With no cases published in the 21st century, this antibody is a rare cause of HDFN. Anti-Go(a) is produced against the Go antigen, a low-frequency Rh antigen found predominantly in African and Puerto Rican populations. Bringing awareness to this rare cause of HDFN may accelerate diagnosis when the physician is faced with non-ABO and non-Rh isoimmunization in these ethnic groups.

## Introduction

Hemolytic disease of the fetus and newborn (HDFN) is an immune-related red blood cell (RBC) disorder in which maternal antibodies attack fetal or newborn RBCs. HDFN can cause significant morbidity and mortality, especially in limited healthcare resource settings. The effects of HDFN range from mild anemia to hydrops fetalis in the fetus and hyperbilirubinemia and kernicterus in the newborn. The two main mechanisms of HDFN are due to ABO incompatibility, which typically causes a less severe form of HDFN, and fetomaternal hemorrhage, in which the most common antigen involved is the Rhesus D antigen that can cause more severe complications of HDFN [1]. Due to the use of anti-D immunoglobulin, the incidence of HDFN due to Rh incompatibility has decreased. However, the incidence of HDFN due to irregular antibodies that are unusual to the group antigens has become more apparent.

The Gonzales (Go) antigen belongs to the Rh blood group system and is known to cause rare irregular autoantibodies. Only three previous known cases of anti-Go have been reported. It was first described in 1956 as a low-frequency Rh antigen found predominantly in people of African descent [2]. In 1967, an antibody was identified in the mother of an infant with hemolytic disease of the newborn (HBN) [3]. It was coined as anti-Gonzales, after the patient’s surname. Anti-Go has been reported to cause HBN. A case report described in 1996 is the only reported incidence of a delayed hemolytic transfusion reaction due to the anti-Go antibody (anti-Go(a)) [4].

The present case explores the rarity of the anti-Go-associated hemolytic disease and its clinical presentation and testing. This information will allow future cases of anti-Go to be readily diagnosed and promptly treated while also emphasizing the importance of complete evaluations during the newborn exam. New information and discovery of women with anti-Go(a) can be followed prenatally with amniotic fluid bilirubin studies, serial antibody titers, and fetal hemoglobin levels to prevent severe complications of HDFN.

## Case presentation

**Figure 1:**
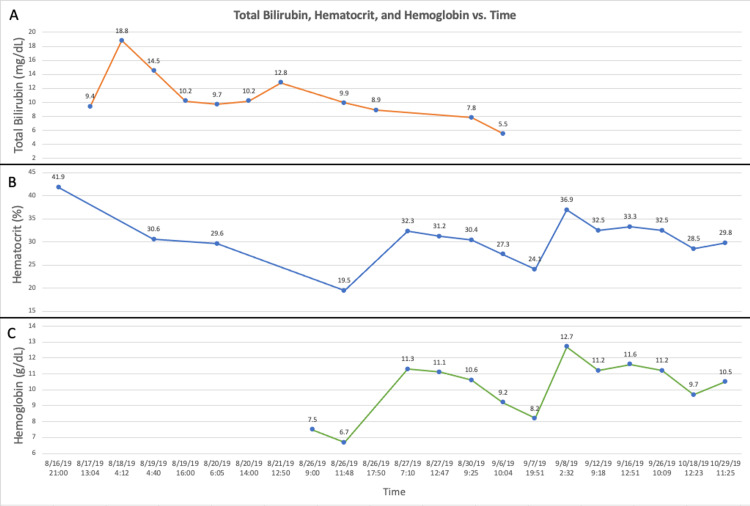
The patient's total bilirubin over time (A), hematocrit over time (B), and hemoglobin over time (C) in a case of hemolytic anemia of the newborn due to the anti-Gonzales antibody

The male patient was born at 38 weeks gestation via cesarean section. He presented with jaundice and a total bilirubin of 9.1 mg/dl at 24 hours of life (Figure 1A). Triple phototherapy was started in the neonatal intensive care unit (NICU), and his total bilirubin jumped to 18.8 mg/dl at 40 hours of life with a hematocrit of 30.6% (Figure 1B). The patient remained on triple phototherapy for three days before being discharged on his fourth day of life (DOL) with a total bilirubin of 10.2 mg/dl and a hematocrit of 29.6%. Further evaluation in the NICU revealed a 3+ direct Coombs test result due to a maternal alloantibody, although the patient and his mother were both RBC O+. Since the Coombs test was positive due to an unknown antigen, the NICU sent an elution and antibody identification test out to a reference lab for further evaluation, but the patient was discharged before the test results were concluded. The patient was subsequently diagnosed with HDN due to non-ABO and non-Rh isoimmunization.

The patient had his pediatric newborn visit on his 11th DOL, where he was found to be in active hemolysis with a total bilirubin of 9.9 mg/dl and a hemoglobin (Hb) level of 6.7 gm/dl (Figure 1C). This required prompt admission and a pRBC transfusion of 15 ml/kg, at the request of his pediatrician. He responded well and was discharged a day later with a Hb of 11.1 gm/dl. The consulting hematologists were able to retrieve the results of the elution and antibody testing and concluded the etiology of his hemolytic anemia to be the anti-Go(a). On further questioning, the mother disclosed that her previous child also required a short course of single-light phototherapy. This revelation provided a consistent clinical picture of this antibody.

During a follow-up appointment with hematology, when the patient was three weeks old, he was found to have a Hb of 9.2 gm/dl and required another pRBC transfusion of 15 ml/kg. It was also revealed that the mother's plasma contained anti-Go(a), only confirming the etiology of her child's hemolytic anemia. Further monitoring of the patient was prompted at that time, with ongoing tracking of his hemoglobin until his antibody titer decreased and he had a negative Coombs test.

By the patient’s 11th week of life, his hemoglobin, reticulocyte count, and total bilirubin levels were stable, and he did not express any physical signs of continued hemolytic anemia. An eluate was then prepared from the patient's red cells and was concluded to be non-reactive with Go(a) red cells. The patient has not required any clinical interventions for anemia since.

## Discussion

HDFN historically has been a feared perinatal/neonatal complication. The most common pathophysiology of this condition involves the RBC surface antigen Rh. The most significant concern arises for Rh- mothers, Rh+ fathers, and their children. The first fetus, who is Rh+, results in the mother becoming sensitized to the paternally derived Rh antigens. Her immune system responds by mounting a defense via antibody production. The first Rh+ child is generally spared from the effects of the alloantibodies circulating in the shared maternal-fetal blood in the placenta. However, subsequent children who are Rh+ have an increased risk of developing HDN [2,5]. 

The classic clinical description is a jaundiced and anemic neonate, with hydrops fetalis being the most significant concern before birth. Maternal-fetal Rh incompatibility is the most common cause of HDN. Fortunately, it has decreased in incidence with the implementation of routine screening and Rh immunoglobulin G prophylaxis [2,5]. However, many other less common antigens can also manifest in severe HDN. The case presented above illustrates the complexity of this rare condition and the challenges of making an accurate diagnosis.

The patient, in this case, displayed the classic signs of hemolytic disease of jaundice and increased bilirubin shortly after birth. Initial investigations revealed a positive direct Coombs test and that both mother and neonate were blood type O, Rh+. The direct Coombs test indicated that the patient's RBCs were coated in antibodies, presumably alloantibodies from the mother. Considering the discussion above regarding HDN, it is clear that traditional Rh incompatibility was not the driver of this neonate's presentation since the mother and the patient were O+. The working diagnosis at this point was non-ABO and non-Rh isoimmunization. The true diagnosis of HDN due to the anti-Go(a) was only discovered after the patient presented to his pediatrician in dire need of a blood transfusion. After that discovery, an essential piece of history revealed by the mother was that her previous pregnancy resulted in a neonate who also needed phototherapy for a shorter duration than the patient required. This confirmed the hematologists' suspicion of the rare anti-Go(a), an irregular antibody still within the Rh system [4]. Further investigation of the mother's plasma did reveal anti-Go(a). 

When considering the entire clinical picture, the patient's presentation, the labs, and the mother's history, the hematologists concluded that the cause of this neonate's HDFN was due to anti-Go(a). The anti-Go(a) and HDFN associations make very few appearances in the literature. To the authors' knowledge, the case presented here is the first to be described in the 21st century [6]. It is essential to bring attention to this rare case because it brings a new understanding of the importance of obtaining a detailed history concerning the mother's previous pregnancies and the neonate's hospital course. This allows early anticipation of potential hemolytic complications and strategic screening for subsequent pregnancies. Educating the family on the potential harms of subsequent pregnancies gives them full patient autonomy to make family planning decisions.

Additionally, parents of certain ethnicities (Black and Puerto Rican for anti-Go) have an increased predisposition to some irregular antibodies [3]. Understanding the unique patterns of the different causes of the HBN allows prompt diagnosis and treatment to increase favorable outcomes for the neonate. Finally, this case should raise awareness of obtaining a hemoglobin level during the pediatrician's newborn assessment, especially when there is clinical concern for jaundice.

## Conclusions

Our case describes a rare incidence of a patient with the irregular antibody, anti-Go(a), resulting in HBN. We describe a case that reports the presentation of HDFN due to anti-Go(a) and how it was only revealed because of the necessary hemoglobin check at the newborn screening. Of the few reports recorded, anti-Go(a) are more common in Black and Puerto Rican individuals. From our review of the current reported cases, we notably found that most individuals were blood type O, Rh+, which may also explain the condition's rarity and warrants further investigation into ABO blood types and their association with hemolytic-causing antigens. This case allows future pediatricians to better identify the clinical features of anti-Go(a) and provide prompt treatment to prevent severe complications of HDFN.
